# Acquisition of Childcare Skills by a Patient With Transient Paraparesis Following Epidural Anesthesia for Cesarean Section

**DOI:** 10.7759/cureus.82036

**Published:** 2025-04-10

**Authors:** Ippei Kitade, Tomoko Yamaguchi, Aya Shirafuji, Yoshinori Endo, Hidetaka Arishima

**Affiliations:** 1 Division of Rehabilitation Medicine, University of Fukui Hospital, Fukui, JPN; 2 Department of Advanced Medicine for Community Healthcare, University of Fukui, Fukui, JPN; 3 Department of Obstetrics and Gynecology, University of Fukui, Fukui, JPN; 4 Department of Neurology, University of Fukui, Fukui, JPN; 5 Department of Neurosurgery, University of Fukui, Fukui, JPN

**Keywords:** cesarean section, childcare skill, epidural anesthesia, rehabilitation, transient paraparesis

## Abstract

For first-time mothers, every aspect of childcare is a new experience, and acquiring these skills can be physically and mentally challenging. It is reasonable to assume that patients with disabilities, including those experiencing lower-limb paraparesis, may face even greater difficulty mastering the physical movements required for childcare while simultaneously striving to regain independence in daily living. We report the case of a 29-year-old woman who developed lower limb paraparesis following an elective caesarean section and describe her recovery in limb function, activities of daily living (ADLs), and childcare skills. She was fully independent in ADLs preoperatively. On postoperative day 1, she experienced lower-limb weakness and sensory disturbances. Physical therapy commenced on day 5, and by week 2, she could perform childcare tasks such as holding, diaper changing, and breastfeeding while seated. By week 3, she was discharged with a wheelchair and walker. She was able to perform ADLs independently by week five. By week 10, she could perform childcare tasks while standing, though lifting and carrying her child remained difficult until week 15 due to physical and psychological factors. Epidural anesthesia after caesarean section facilitates early mobilization but may cause complications. This case highlights the need for standardized childcare assessments and structured rehabilitation programs.

## Introduction

Epidural anesthesia is a highly effective method for pain relief, commonly used to alleviate severe postoperative pain, and facilitates early mobilization and rehabilitation, ultimately promoting functional recovery. However, it carries risks of complications, including nausea, vomiting, and hypotension. von Hösslin et al. [1] reported severe complications, such as epidural abscess, permanent nerve damage, cardiac arrest, catheter rupture with retained material, and temporary nerve impairment. Similarly, Sakura et al. documented a case of temporary lower-limb paralysis following a cesarean section [2].

For first-time mothers, every aspect of childcare is a new experience, and acquiring these skills can be physically and mentally challenging. It is reasonable to assume that patients with disabilities, including those experiencing lower-limb paralysis, may face even greater difficulty mastering the physical movements required for childcare while simultaneously striving to regain independence in daily living. Honey et al. identified difficult childcare tasks in patients with disabilities, including lifting, carrying, transportation, and bathing [3]. Although rehabilitation programs aimed at reintegrating patients with various disabilities into daily life have been documented, there is a lack of research on acute rehabilitation programs specifically designed to support the acquisition of childcare skills.

Here, we describe the progression of lower-limb function recovery, activities of daily living (ADLs) and childcare skill acquisition in a patient who developed transient paraparesis as a complication of epidural anesthesia following an elective caesarean section. After undergoing rehabilitation, she achieved a high level of functional recovery, independence in ADLs, and proficiency in childcare skills.

## Case presentation

**Figure 1:**
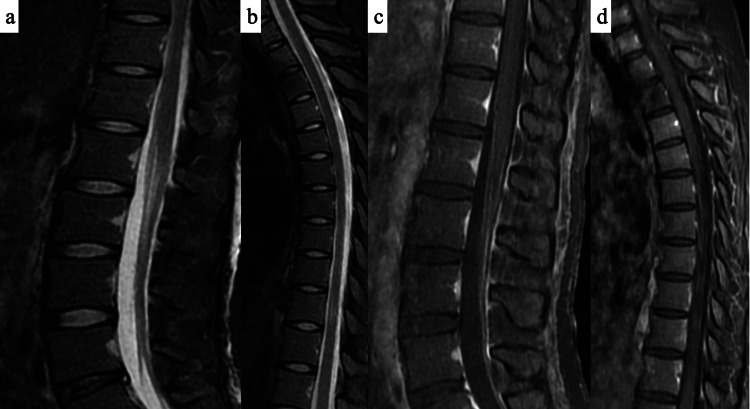
Magnetic resonance imaging findings at three days postoperatively A T2-weighted magnetic resonance imaging (MRI) scan showed no significant abnormal signals in the vertebrae or intervertebral discs (a: lumbar, b: thoracic). A contrast-enhanced MRI scan observed no abnormal enhancement or contrast agent leakage into the epidural space (c: lumbar, d: thoracic).

A 29-year-old woman (55.9 kg, 157.5 cm) was admitted for an elective cesarean section due to breech presentation. She was fully independent in ADLs before pregnancy and could ambulate independently preoperatively. She had no significant medical history or abnormal preoperative laboratory results. The caesarean section was uneventful, and she delivered successfully. However, that evening, she developed a fever above 39°C, prompting the initiation of cefmetazole sodium. The following day, she experienced diminished superficial sensations (touch, pressure, temperature, and pain) in both lower extremities, accompanied by weakness upon waking. Consequently, the attending physician reduced the epidural fentanyl citrate dose and monitored her condition. By postoperative day 2, her sensory deficits and lower limb paralysis had worsened, leading to the discontinuation of epidural anesthesia. On postoperative day 3, magnetic resonance imaging (MRI) revealed no abnormal vertebral or intervertebral disc signals (Figures 1a, 1b). Contrast-enhanced MRI showed no abnormal enhancement or epidural contrast agent leakage (Figures 1c, 1d). The hospital safety committee confirmed adherence to standard anesthetic protocols. Additional tests ruled out conditions such as Guillain-Barré syndrome.

Rehabilitation began on postoperative day 5. Physical findings are summarized in Table 1. The Medical Research Council (MRC) scale for muscle strength indicated scores of one for hip flexors, two for knee extensors, and three for foot dorsiflexors bilaterally, with pronounced proximal muscle weakness. Sensory examination revealed numbness and hypoesthesia from T6 to L5, most severe proximally. The patient required assistance to stand but could sit independently, albeit with difficulty (International Stoke Mandeville Wheelchair Sports Federation (ISMWSF) classification: fair [4]). Paralysis was classified as American Spinal Injury Association (ASIA) Impairment Scale grade C, and the Functional Independence Measure (FIM) score was 78/126. She also reported pain at the cesarean incision site. Her primary goals were regaining independence in daily living and safely performing childcare tasks.

**Table 1:** Progress of physical findings and childcare tasks MRC: Medical Research Council; the MRC scale for muscle strength is scored on a scale of 0 (no contraction) to five (normal). The score was defined as the sum of scores from six muscles in the upper and lower extremities on both sides, so that the score ranged from 0 to 60. Rt.: right; Lt.: left; ASIA: American Spinal Injury Association; ISMWSF: International Stoke Mandeville Wheelchair Sports Federation; FIM: Functional Independence Measure

Parameters		Five days	One week	Two weeks	Five weeks	10 weeks	15 weeks	17 weeks
MRC scale (points)	Side of the body	42	48	51	54	54	54	58
Shoulder abductors	Rt.	5	5	5	5	5	5	5
	Lt.	5	5	5	5	5	5	5
Elbow flexors	Rt.	5	5	5	5	5	5	5
	Lt.	5	5	5	5	5	5	5
Wrist extensors	Rt.	5	5	5	5	5	5	5
	Lt.	5	5	5	5	5	5	5
Hip flexors	Rt.	1	2	2	3	3	3	4
	Lt.	1	2	2	3	3	3	4
Knee extensors	Rt.	2	3	3	4	4	4	5
	Lt.	2	3	4	4	4	4	5
Foot dorsiflexors	Rt.	3	4	5	5	5	5	5
	Lt.	3	4	5	5	5	5	5
Numbness/ Hypesthesia	Rt.	T6-L5	T6-L5	T9-L5	T9-L3	T11-L2	T11-L2	T11-L2
	Lt.	T6-L5	T6-L5	T9-L5	T9-L5	T11-L3	T11-L3	T11-L2
ASIA impairment scale		C	D	D	E	E	E	E
ISMWSF classification		Fair	Good	Normal	Normal	Normal	Normal	Normal
Gait ability		Impossible	Possible (with a walker (100m); With a cane (10m))	Possible (With a walker (200m); With a cane (50m))	Independent ambulation	Independent ambulation	Independent ambulation	Independent ambulation
									FIM (points)		78	−	114	124	126	126	126
																		Childcare skills								
Holding a child		Impossible	Possible (sitting without back support)	Possible (sitting without back support)	Possible (sitting without back support)	Possible (standing)	Independent	Independent
									Lifting a child		Impossible	Impossible	Possible (sitting without back support)	Possible (sitting without back support)	Possible (standing)	Independent	Independent
Carrying or transporting a child		Impossible	Impossible	Impossible	Impossible	Possible (on flat surfaces only)	Possible (with steps and slopes)	Independent									
									Bathing		Impossible	Impossible	Impossible	Impossible	Possible	Independent	Independent
Feeding		Possible (with bed elevation)	Possible (sitting without back support)	Possible (sitting without back support)	Possible (standing)	Independent	Independent	Independent									
									Changing diapers		Impossible	Possible (sitting without back support)	Possible (sitting without back support)	Possible (standing)	Independent	Independent	Independent

Rehabilitation therapy (physical therapy) was administered for 40 minutes daily, five times a week. Initially, exercises focused on achieving a seated posture without back support. Strength training and standing balance exercises were introduced to promote independent ambulation. While she managed to maintain a seated posture unaided, her balance remained weak in response to external stimuli (ISMWSF classification: fair [4]). With progressive training, her standing balance improved, allowing her to maintain an upright posture for approximately five minutes using a forearm-supported walker. By one week postoperatively, the focus shifted to seated posture, standing, and gait exercises. As a result, her sitting balance improved (ISMWSF classification: good [4]), enabling her to sit unaided and breastfeed in a seated position. She could walk along the bed using rails, transfer independently to a wheelchair, walk approximately 100 m with a forearm-supported walker, and 10 m with a T-cane (ASIA impairment scale grade D).

From postoperative week 2, standing and gait exercises focused on muscle strengthening. She walked approximately 200 m with a forearm-supported walker and along rails. Basic seated ADLs, such as transferring and feeding, were performed independently; however, standing to hold her baby remained challenging due to unsteadiness and knee buckling. At postoperative week 3, she was discharged using a walker and a wheelchair. Her husband took four months of paternity leave to assist with childcare and patient care. After discharge, he managed lifting, carrying, transportation, and bathing, while she handled diaper changing and breastfeeding while seated. From postoperative week 4, she participated in weekly 60-minute physical therapy sessions. The rehabilitation program included leg and trunk stretching, muscle strengthening with aerobic activity, side steps, coordination exercises (crawling, kneeling), floor-to-stand training, and advanced gait exercises with weights.

By postoperative week 5, she attained supervised independent ambulation, classified as ASIA impairment scale grade E. The MRC scale for muscle strength indicated scores of three for hip flexors, four for knee extensors, and five for foot dorsiflexors bilaterally. She could also walk outdoors and push a stroller while carrying her child. At 10 weeks, she regained independence in ADLs without requiring a seated position (FIM score: 126/126). She resumed driving and could hold and carry her child while walking. By 15 weeks, she could ascend and descend stairs while carrying her child. With her husband's paternity leave ending at this period, she managed solo parenting during working hours.

At the final 17-week follow-up evaluation, the MRC scale for muscle strength revealed scores of four for hip flexors and five for other lower extremity muscles, indicating an improvement in muscle strength. No decline in ADLs or childcare was observed. Numbness and hypoesthesia between T11 and L2 levels had diminished. The scheduled follow-up was completed.

## Discussion

Complications of epidural anesthesia during surgical procedures and postoperative pain management can include neurological damage, although the incidence is extremely low [5, 6]. Typical symptoms include transient neurological deficits, such as cauda equina syndrome or nerve root irritation, which manifest as sensory and motor disturbances in the affected nerve distributions, while permanent nerve damage is rare [1, 5, 6]. Contributing factors include mechanical damage, neurotoxicity, and pressure from hematomas. Additionally, some patients may have underlying predispositions that increase their susceptibility to these complications [2, 6-10].

In this case, the patient developed transient neurological symptoms following a caesarean section under epidural anesthesia. Since MRI findings showed no contrast leakage into the epidural space, we hypothesized that the symptoms were caused by the neurotoxicity of the anesthetic or an underlying predisposition. Although the patient returned home after three weeks with assistive devices, she had not fully regained her ADLs or child-rearing functions. Reports on transient neurological impairment following epidural anesthesia for cesarean section are scarce (Table 2) [2, 6-10]. In documented cases, symptoms typically occur within two days postoperatively, with motor function recovering over several weeks. However, sensory abnormalities, such as numbness, often persist beyond six months [2, 6-10]. Treatment strategies include diclofenac sodium suppositories [2], high-dose corticosteroid therapy [6, 7, 10], and rehabilitation [7-9]. Chen et al. [9] explored hyperbaric oxygen therapy alongside traditional Chinese medicine and acupuncture; however, their patient was ultimately determined not to have a transient neurological disorder four weeks postoperatively. Although rehabilitation has been reported, details on the specific programs, progress, or the acquisition of childcare functions are lacking. To our knowledge, this is the first case report detailing the specific contents of the acute rehabilitation program and childcare skill acquisition in a patient with transient paraparesis following epidural anesthesia for a caesarean section.

**Table 2:** Transient neurological impairment following epidural anesthesia for cesarean section -: no; + yes; ↑: improved; ↓: reduced

Author, year	Age (years)	Onset (after surgery)	Symptoms	Treatment	Rehabilitation	Final outcome	Duration of recovery
Kitazaki et al., 2020 [6]	31	2 days	Lower limb paralysis	High-dose corticosteroid therapy	−	Gait possible↑	15 days
			Hypesthesia in the lower limbs			Hypesthesia↓	Residual
Sakura et al., 2002 [2]	28	2 days	Thigh and perianal pain	Diclofenac sodium suppositories	−	Pain↓	7-11days
			Sacral region numbness			Numbness↓	5-6 months
Marinho et al., 2021 [7]	33	48 hours	Non-ambulatory	High-dose corticosteroid therapy	+	Gait possible↑	No recorded
			Perianal hypesthesia			Perianal hypesthesia	Residual
Srifakioglu et al., 2013 [8]	39	1 day	Lower limb paralysis	-	+	Gait possible↑	A few weeks
			Non-ambulatory				
			Hypesthesia in the lower limbs			Hypesthesia↓	Residual
Chen et al., 2015 [9]	29	30 hours	Non-ambulatory	Traditional Chinese medicine	+	Gait possible↑	a few days
			Right drop foot	Hyperbaric oxygen therapy		Right drop foot	Residual
			Hypesthesia in the lower limbs	Acupuncture		Hypesthesia↓	4 weeks
Takasu et al., 2010 [10]	29	48 hours	Lower limb paralysis	High-dose corticosteroid therapy	−	Gait possible↑	8 weeks
			Non-ambulatory				
			Hypesthesia in the lower limbs			Hypesthesia	Residual

Honey et al. [3] conducted a survey on childcare tasks among physically disabled patients, revealing that the most difficult periods were 0 to one year (40%) and one to two years (45%). The most commonly reported difficulties included bathing, feeding, holding, lifting, and carrying or transporting, emphasising the need for healthcare providers to address these factors [3, 11, 12]. Additionally, rehabilitation priorities focused on carrying or transporting, holding or lifting, and bathing activities essential for independent childcare. Our rehabilitation program not only aimed to improve physical function and ADLs but also incorporated motion training related to childcare activities. The patient regained gait and ADL function within eight to 10 weeks postoperatively, consistent with previous studies. However, mastering childcare skills such as bathing, feeding, holding, lifting, and carrying or transporting required up to 15 weeks, likely due to a combination of physical factors (recovery from transient paraparesis) and psychological factors (fear of dropping the child).

## Conclusions

Although ADLs have been extensively studied, no standardised tools exist for assessing childcare skills. We propose the development of standardised and quantitative assessments applicable to both disabled and non-disabled parents, integrating prenatal and postnatal perspectives. Mastering childcare skills required up to 15 weeks, likely due to a combination of physical factors and psychological factors.
